# Ablation compared with excision in the surgical management of peritoneal endometriosis: a retrospective study of pain, re-operation, and pregnancy outcomes

**DOI:** 10.1007/s00404-026-08331-4

**Published:** 2026-02-02

**Authors:** Thomas Kolben, Lennard Schröder, Charlene Kaiser-Rix, Sven Mahner, Susanne Beyer, Lucia Ehmann, Bastian Czogalla, Christina Seifert, Franziska Ganster, Bernd Kost, Alexander Burges, Fabian Trillsch, Simon Keckstein

**Affiliations:** 1 Department of Obstetrics and Gynecology, LMU University Hospital, LMU Munich, Munich, Germany; 2 Gyn-Munich gynecologic surgery, Heidemannstr. 5b, 80939 Munich, Germany; 3 Gynäkologische Praxisklinik München, München MVZ GmbH, Munich, Germany

**Keywords:** Endometriosis, Laparoscopy, Excision, Ablation, Pain, Hormonal therapy, Fertility, Re-operation

## Abstract

**Objective:**

The study aimed to evaluate the long-term outcomes of surgical management in patients with peritoneal endometriosis, focusing on postoperative pain trajectories, re-operation rates, fertility outcomes, and the potential influence of hormone therapy.

**Methods:**

This retrospective study included 67 patients with histologically confirmed peritoneal endometriosis who underwent laparoscopic surgery. Surgical management consisted of excision in 62.7% of cases, ablation using bipolar coagulation in 13.4%, and a combination of both techniques in 23.9%. Pain symptoms (dysmenorrhea, dyspareunia, and chronic pelvic pain) were assessed preoperatively at 6 and 12 months, and at a median follow-up of 42 months. Associations between surgical technique, postoperative hormone therapy, and pain outcomes over time were analyzed using mixed linear models.

**Results:**

Both excision and ablation were associated with significant and sustained reductions in pain symptoms over time. Dysmenorrhea showed improvement postoperatively, with additional benefit observed in patients receiving hormonal therapy. The type of surgery had no significant effect on dysmenorrhea. Dyspareunia and chronic pelvic pain also improved during follow-up, independent of surgical technique or hormone use. Re-operation was required in 17.9% of cases, with no difference between excision and ablation. Among the 27 patients who wished to conceive, 62.9% achieved pregnancy postoperatively, irrespective of surgical approach.

**Conclusions:**

Both excision and ablation using bipolar coagulation are effective surgical options for peritoneal endometriosis, leading to long-term pain relief and favorable fertility outcomes. Postoperative hormone therapy appears to enhance pain control, particularly for dysmenorrhea. Overall, symptom improvement was more strongly associated with time since surgery than with the specific surgical technique, supporting individualized and multimodal treatment strategies.

**Supplementary Information:**

The online version contains supplementary material available at 10.1007/s00404-026-08331-4.

## What does this study add to the clinical work

**Table:** 

In women with peritoneal endometriosis, excision and ablation showed similar long–term results for pain relief and pregnancy outcomes. This supports an individualized surgical approach in which postoperative management and patient factors may be more important than the choice of surgical technique itself.	

## Introduction

Endometriosis is a chronic, estrogen-dependent inflammatory disease characterized by the presence of endometrial-like tissue outside the uterus [1]. It affects approximately 10% of women of reproductive age and up to 50% of women with infertility or chronic pelvic pain [2]. Although its pathogenesis is considered multifactorial and remains incompletely understood [3–6], endometriosis most commonly involves the peritoneum, ovaries, and fallopian tubes, with potential infiltration of adjacent pelvic organs [7]. Given its chronic and heterogeneous nature, effective management strategies—particularly surgical treatment of peritoneal disease—are essential to improve pain, reduce recurrence, and preserve fertility.

Endometriosis is associated with a wide range of symptoms, often leading to a significant delay in diagnosis, with an average of 7 to 10 years from symptom onset [8]. The most common clinical manifestations include dysmenorrhea, chronic pelvic pain, dyspareunia, dyschezia, and infertility [9]. However, the severity of symptoms does not always correlate with the extent of the disease, making diagnosis and treatment difficult. In addition to its physical impact, endometriosis has a profound effect on mental health, work productivity, sexual function, and overall quality of life [10].

Diagnosis of endometriosis remains a challenge. While imaging techniques such as transvaginal ultrasound and MRI can detect some forms of the disease, superficial peritoneal lesions often go unnoticed [11]. Despite advances in imaging, clinical assessment, and biomarker research, laparoscopy with histology remains the reference standard, though diagnosis can often be established without surgery, especially in ovarian endometrioma and deep endometriosis [12]. Staging systems such as the revised American Society for Reproductive Medicine (ASRM) classification and the #Enzian system are commonly used for the staging of superficial and deep infiltrating endometriosis (DIE), but they do not always predict symptom severity or treatment outcomes, highlighting the need for individualized management approaches [13, 14].

Surgical treatment is considered in patients with persistent symptoms despite medical therapy, large ovarian endometriomas, destruction and dysfunction of organs or infertility that has not responded to conservative treatment [15, 16]. The primary surgical approaches include excision, where endometriotic lesions are completely removed, and ablation, where lesions are thermally or laser coagulated [17]. Excision is often preferred because of its potential for long-term symptom relief and lower recurrence rates, while ablation is a less invasive but potentially less effective alternative as the depth of some lesions may be underestimated. In some cases, a combination of the two techniques is used [18]. Postoperatively, hormone therapy is often recommended to suppress residual disease and delay recurrence, but its effectiveness varies according to individual patient factors [19, 20].

Despite ongoing research, the optimal surgical approach for peritoneal endometriosis remains controversial, with limited consensus on long-term outcomes in terms of pain recurrence, reoperation rates, and fertility. While there is data from RCT randomized controlled trials assessing the optimal surgical approach for peritoneal endometriosis, the studies' observational period is limited to 6 to 12 months, often also focusing on pain modalities without looking at the aspects of long-term pain relief, reoperation rates, and pregnancy outcomes after surgery [21, 22]. Therefore, this study presents a retrospective analysis of patients undergoing surgery for peritoneal endometriosis, evaluating the aspects mentioned above. In addition, the study examines the impact of post-operative hormone therapy on symptom control and disease progression. By analyzing a well-defined cohort, this study aims to contribute to the ongoing discussion on optimizing surgical strategies and postoperative management for women with peritoneal endometriosis.

## Material and methods

This study is a retrospective analysis of women diagnosed with peritoneal endometriosis who underwent surgical treatment at a university hospital between June 2016 and December 2021. Women with deep infiltrating endometriosis or endometriomas were excluded. The dataset includes 67 women with complete records of surgical outcomes, pain scores, reoperation rates, and pregnancy outcomes. Data collection focused on preoperative characteristics, intraoperative findings, postoperative pain evolution, and subsequent fertility outcomes. The study was approved by the ethical committee of the University of Munich (project-nr. 22–1035, on the 11 January 2023).

Patients were categorized according to surgical technique: excision or ablation using bipolar coagulation. Women who underwent both excision and ablation were included in the ablation group for statistical analysis. Women treated with both excision and ablation were categorized within the ablation group to ensure a conservative comparison, based on the assumption that potential recurrence associated with ablation would not be attributed to the more radical excision approach.

Moreover, grouping these patients with the ablation cohort allowed for a conservative comparison of surgical techniques while avoiding the creation of a small, heterogeneous third group with limited statistical power. Intraoperative staging followed the #Enzian staging system, focusing on the peritoneal compartment (P). Peritoneal endometriosis was staged based on the largest diameter of visible peritoneal lesions assessed during laparoscopy: P1 for lesions < 3 cm, P2 for lesions ≥ 3 cm, and P3 for lesions ≥ 7 cm. Staging was performed intraoperatively by the operating surgeon using direct visual inspection and surgical documentation, and postoperatively the images of surgery were assessed in cases where #Enzian-scores were not documented (some patients were operated on before the #Enzian classification system was published and applied in our clinic). Pain was assessed using numeric rating scale (NRS) scores preoperatively, at six months, at 12 months, and at final follow-up during retrospective data collection. The use of postoperative hormone therapy, including gestagen-only pills, combined oral contraceptives, and hormonal intrauterine pessaries, was also documented at each time point.

Postoperative outcomes were analyzed for recurrent pain (dysmenorrhea, dyspareunia, chronic pelvic pain), need for re-operation, and subsequent pregnancy rates. Statistical comparisons were made using chi-squared tests for categorical variables, independent t-tests for continuous variables, and linear mixed effects models to assess the progression of pain scores over time. A significance level of *p* < 0.05 was considered statistically significant. Data processing and statistical analysis were conducted using R [23], version 4.4.2, and its corresponding libraries, lme4 [24] and lmerTest [25], to fit and evaluate random effects models.

## Results

### Patient characteristics and surgical approaches

The study involved 67 patients with a median age of 29 (IQR; 25–34) years. The primary reason for consultation was endometriosis-related pain (60 patients, 89.5%), followed by infertility (20 patients, 29.8%), and in one patient as an incidental finding (1.4%). The median duration of infertility was 28 (IQR: 12–36) months.

Patients underwent different surgical techniques: excision (42 patients, 62.7%), ablation (9 patients, 13.4%), and combined excision and ablation (16 patients, 23.9%). The average hospital stay was 2.1 ± 0.8 days. The median interval between the operation and the retrospective inquiry was 42 (IQR: 36 – 52) months. The stages of peritoneal endometriosis according to the #Enzian classification were as follows: P1: 52; P2: 15; P3: 0. There was no significant difference regarding the severity of the endometriosis and the treatment method used (*p* = 0.24) (Table 1).

**Table 1:** Characteristics of the study population. Numerical variables are represented by their median and IQR, while frequencies and relative frequencies are indicated in brackets

Characteristics of the study population	
Number of patients	67
Age at surgery in years	29 (IQR: 25—34)
Time between surgery and inquiry in months	42 (IQR: 36 – 52)
Patients with previous pregnancies	7 (10.4%)
Patients with excision of lesions	42 (62.7%)
Patients with ablation of lesions	9 (13.4%)
Patients with excision and ablation of lesions	16 (23.9%)
Patients with preoperative hormonal therapy	19 (28.3%)
Patients with postoperative hormonal therapy	32 (47.7%)
Patients with Re-operations	12 (17.9%)

### Pain outcomes and hormone therapy

Preoperatively, 19 (28.3%) patients already were under hormonal treatment. Postoperatively, hormonal therapy was recommended to 53 (79.1%) patients, and 32 (47.7%) patients followed this recommendation (Table. 2).

**Table 2:** Distribution of pain severity across four categories (0, 1–3, 4–7, and 8–10) on the Numeric Rating Scale (NRS) for three types of pain: dysmenorrhea, dyspareunia, and chronic pelvic pain

Pain modality	Time point	NRS 0	NRS 1–3	NRS 4–7	NRS 8–10
Dysmenorrhea	Surgery	12 (17.9%)	1 (1.5%)	15 (22.4%)	39 (58.2%)
	6 Month	30 (44.8%)	15 (22.4%)	16 (23.9%)	6 (9.0%)
	12 Month	26 (38.8%)	12 (17.9%)	17 (25.4%)	12 (17.9%)
	Last Follow-Up	33 (49.3%)	15 (22.4%)	14 (20.9%)	5 (7.5%)
Dyspareunia	Surgery	58 (86.6%)	2 (3.0%)	4 (6.0%)	3 (4.5%)
	6 Month	62 (92.5%)	2 (3.0%)	1 (1.5%)	2 (3.0%)
	12 Month	62 (92.5%)	1 (1.5%)	3 (4.5%)	1 (1.5%)
	Last Follow-Up	63 (94.0%)	2 (3.0%)	1 (1.5%)	1 (1.5%)
Chronic Pelvic Pain	Surgery	48 (71.6%)	1 (1.5%)	5 (7.5%)	13 (19.4%)
	6 Month	56 (83.6%)	1 (1.5%)	7 (10.4%)	3 (4.5%)
	12 Month	56 (83.6%)	1 (1.5%)	6 (9.0%)	4 (6.0%)
	Last Follow-Up	59 (88.1%)	1 (1.5%)	4 (6.0%)	3 (4.5%)

Both the absolute number and the corresponding percentage are reported for each category. Data are presented for four time points: at the time of surgery; 6 months postoperatively; 12 months postoperatively; and at the time of the retrospective assessment (“Last Follow-Up”)

We used mixed linear models to investigate the potential influence of surgical technique, hormonal therapy, and the time since surgery on different pain modalities. The regression coefficient (β) quantifies the direction and magnitude of the change in NRS pain scores associated with each predictor. A negative β value for hormonal therapy indicates that such therapy is associated with a reduction in pain, that is, an improvement in the NRS score by a certain number of points. Surgical technique was coded with ablation as the reference category. Therefore, the coefficient for excision reflects the direct effect of undergoing excisional surgery compared to ablation. Positive or negative values indicate an increase or decrease in NRS scores, respectively. Additionally, time since surgery (in months) was modeled as a continuous variable, with each month contributing to a further change in NRS score in the direction indicated by the sign of its β coefficient.

For dysmenorrhea, we found a significant reduction in pain in patients receiving hormonal therapy (*p* = 0.017, *β* = − 1.13, 95% CI [− 2.06, − 0.20]), as well as a significant association with the time elapsed since surgery (*p* < 0.001, *β* = − 0.06, 95% CI [− 0.08, − 0.04]). The type of surgery did not have a significant impact on pain development (*p* = 0.40, *β* = − 0.53, 95% CI [− 1.75, 0.70]).

For dyspareunia, only the time since surgery showed a statistically significant improvement in pain symptoms (*p* = 0.04, *β* = 0.0062, 95% CI [− 0.01, 0.00]). Neither the surgical technique (*p* = 0.30, *β* = 0.37, 95% CI [− 0.34, 1.08]) nor hormonal therapy (*p* = 0.16, *β* = 0.22, 95% CI [− 0.09, 0.54]) was associated with statistically significant effects.

Regarding chronic pelvic pain, symptom improvement over time was also statistically significant (*p* < 0.001, *β* = − 0.02, 95% CI [− 0.03, − 0.01]). However, neither the type of surgery (*p* = 0.05, *β* = 1.10, 95% CI [− 0.01, 2.20]) nor hormonal therapy (*p* = 0.32, *β* = 0.33, 95% CI [− 0.97, 0.32]) showed a statistically significant effect.

The complete mixed linear models are presented in Supplementary Table 1.

### Re-operation rates and fertility outcomes

A further surgical intervention was required in 12 of all cases (17.9%), including nine patients in whom endometriosis had been excised and three in whom it had been ablated. There was no statistically significant difference between the surgical techniques (*p* = 0.51). The type and reason for another surgery are shown in Supplementary Table 2.

**Fig. 1:**
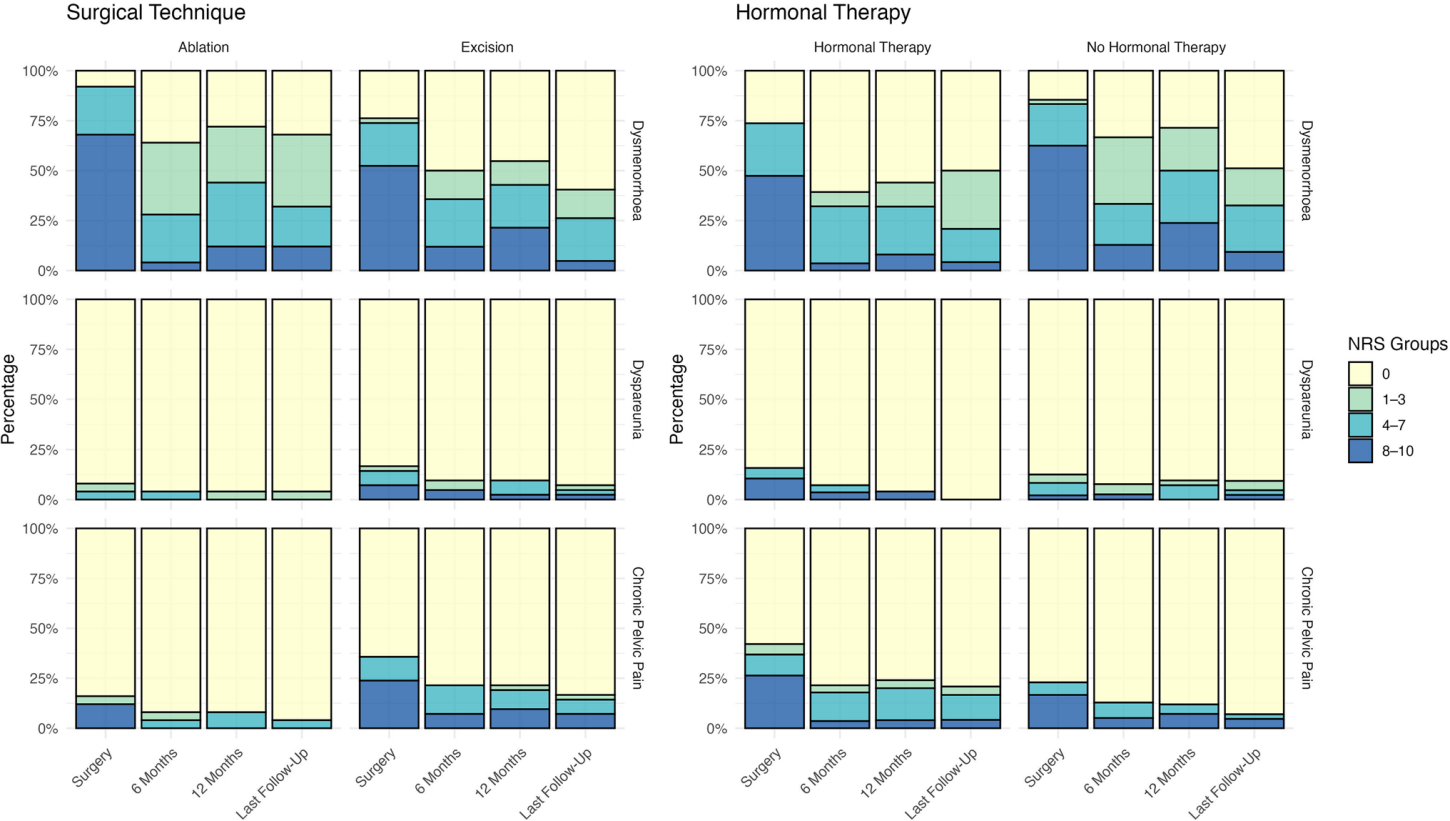
The stacked bar charts show the distribution of Numeric Rating Scale (NRS) scores for dysmenorrhea, dyspareunia, and chronic pelvic pain at the time of surgery, and at three postoperative time points: 6 months, 12 months, and the time of the retrospective survey, plotted as “Last Follow-Up”. NRS scores were grouped into four categories: 0, 1–3, 4–7, and 8–10. The left-hand panel shows the distribution stratified by surgical technique (ablation versus excision), and the right-hand panel shows the distribution stratified by concurrent hormonal therapy (no hormonal therapy versus hormonal therapy). The y-axis shows the proportion of patients in each NRS category at each time point

Of the 27 women who expressed a desire to conceive immediately after surgery, 17 (62.9%) became pregnant. The median time to conception was 15 (IQR: 3.75–24) months. There was no statistically significant difference in conception rates between the surgical modalities (*p* = 0.39). Specifically, 8 of 14 women (57.1%) in the ablation group and 9 of 13 women (69.2%) in the excision group became pregnant (Fig. 1).

## Discussion

This retrospective analysis of 67 patients with peritoneal endometriosis demonstrated that both excision and ablation were associated with significant and sustained improvements in pain symptoms over long-term follow-up. Reductions were observed across all assessed pain domains, including dysmenorrhea, dyspareunia, and chronic pelvic pain, without significant differences between surgical techniques. Re-operation rates were comparable between excision and ablation, and postoperative fertility outcomes were favorable, with nearly two-thirds of patients wishing to conceive achieving pregnancy, independent of the surgical approach. In addition, postoperative hormone therapy was associated with improved pain control, particularly for dysmenorrhea. Despite its retrospective design and limited sample size, this study provides insights into surgical outcomes after excision and ablation for peritoneal endometriosis and emphasizes the need for prospective comparative studies [26].

The findings of the present study are consistent with the Cochrane review by Bafort et al. [27], which demonstrated moderate-quality evidence for improved viable intrauterine pregnancy rates following laparoscopic surgery compared with diagnostic laparoscopy alone in women with minimal to mild endometriosis, but reported limited evidence when comparing excision and ablation for pain relief. Wright et al. evaluated a cohort of 24 patients with a follow-up period of 6 months. No statistically significant differences were observed between excision and ablation [22]. Similarly, Healey et al. compared both surgical approaches regarding symptom improvement. After a follow-up of one year, no clinically relevant differences in endometriosis-associated symptoms were detected depending on the chosen surgical method [21]. Similarly, in our cohort of patients with isolated peritoneal endometriosis, no superiority of excision over ablation was observed regarding pain reduction, re-operation rates, or fertility outcomes.

Although excision is often favored due to theoretical advantages such as complete lesion removal and histological confirmation, the available evidence—including both the Cochrane review and the present study—does not conclusively demonstrate superior clinical outcomes in patients with minimal or mild peritoneal disease. These findings emphasize the importance of individualized surgical decision-making that considers lesion phenotype, symptom burden, fertility goals, surgical expertise, and postoperative management rather than assuming universal superiority of one technique over the other [28]. An additional challenge in interpreting comparative outcomes lies in the term “ablation” itself, which includes a heterogeneous group of surgical techniques such as laser vaporization, argon plasma coagulation, and electrocoagulation and, although derived from the Latin *ablatio* meaning “removal,” is used inconsistently in modern surgery because many techniques involve tissue destruction rather than excision. These approaches differ in tissue penetration depth, lateral thermal spread, and the potential for residual disease and can make it difficult to clearly compare excision and ablation, particularly in retrospective studies [29].

Pain outcomes improved over time regardless of surgical technique. Dysmenorrhea was the most prevalent preoperative symptom and improved progressively after surgery. Postoperative hormone therapy was significantly associated with additional improvement in dysmenorrhea, supporting its role in long-term symptom control, whereas the choice of surgical technique—excision or ablation—did not significantly influence dysmenorrhea outcomes. These findings align with previous evidence indicating that surgical removal or ablation of endometriotic lesions, when followed by hormonal suppression, effectively improves cyclical pain [30]. In line with this, Vercellini et al. reported a nearly 50% reduction in the risk of recurrent dysmenorrhea among women using postoperative combined oral contraceptives or progestins [31].

Dyspareunia was less frequent preoperatively and showed only modest improvement following surgery. Neither surgical technique nor postoperative hormone therapy was significantly associated with dyspareunia outcomes; instead, symptom improvement was primarily related to time since surgery. The gradual improvement over time supports the benefit of multimodal treatment strategies, such as pelvic floor physiotherapy and behavioral interventions, particularly in patients with persistent symptoms [32]. As described by Abbott et al. [33], while the effectiveness of excision in reducing dysmenorrhea is well-established, its impact on dyspareunia appears less predictable, potentially due to mechanisms such as central sensitization leading to neuropathic pain [34, 35]. Taken together, these observations reflect the complexity of endometriosis-associated pain and reinforce the importance of individualized, multimodal management approaches [36].

Postoperative hormonal therapy was recommended to patients not seeking immediate conception; however, adherence in this cohort was limited, with fewer than half of eligible patients continuing treatment. Despite this, hormonal suppression was associated with a significant reduction in dysmenorrhea and showed favorable, although not statistically significant, trends toward reduced chronic pelvic pain and lower re-operation rates [37, 38]. The observed suboptimal adherence highlights the importance of comprehensive patient counseling regarding the long-term benefits of hormonal suppression, balanced against potential adverse effects and individual preferences. Hormonal therapy is not appropriate for all patients, particularly those actively attempting conception, and long-term treatment remains challenging due to side effects and issues of tolerability. These considerations underscore the need for individualized postoperative management strategies that align therapeutic effectiveness with patient-centered decision-making [39].

The re-operation rate in the cohort was found to be relatively low (17.9%), and no differences were associated between excision and ablation rates, suggesting comparable long-term surgical durability for both techniques in patients with peritoneal endometriosis.

In comparison, Roman et al. [40] reported re-operation rates of up to 28% over a 10-year follow-up in a large cohort of 1,092 patients, most of whom had more advanced disease, including deep infiltrative endometriosis. Furthermore, in that cohort, the preservation of the uterus was associated with an elevated risk of re-operation. The lower re-operation rate observed in our study is therefore likely attributable to the restriction to peritoneal disease and may further reflect the influence of postoperative management strategies, including hormonal suppression. Given the chronic and recurrent nature of endometriosis, long-term follow-up studies are essential to better assess recurrence and re-operation risks associated with each surgical technique.

Conversely, no substantial differences were associated in fertility outcomes between the two techniques. Of the 27 patients who expressed an immediate desire for conception postoperatively, two-thirds became pregnant, with a median time to conception of 15 months. These data underline the reproductive potential after surgical treatment, independent of the surgical modality. This finding is consistent with previous research indicating that surgical treatment for peritoneal endometriosis can improve spontaneous conception rates [22, 23]. However, the role of surgery in fertility remains a subject of debate, as assisted reproductive technologies (ARTs), such as in vitro fertilization (IVF), may be necessary in cases of infertility that do not respond to other treatment options [23]. Consistent with the Cochrane review by Bafort et al., moderate-quality evidence supports laparoscopic surgery in improving pregnancy rates in women with minimal to mild endometriosis [27].

This study has several limitations that should be considered when interpreting the results. As a retrospective analysis, it is inherently subject to selection bias, incomplete documentation, and residual confounding, which may have influenced both treatment allocation and outcomes. Surgeries were performed by different surgeons, and decisions about the surgical technique were based on individual clinical judgment. Together with the absence of standardized perioperative and postoperative protocols, this may have led to variability in surgical management and influenced both short- and long-term outcomes. There is a risk of indication bias, as patients with more extensive or deeply infiltrating lesions may have been more likely to undergo excision, whereas milder cases might have been treated with ablation. This non-randomized allocation could have introduced systematic differences between groups and compromised the comparability of outcomes. Finally, the relatively small sample size, especially for subgroups such as those seeking pregnancy or undergoing reoperation, limits the statistical power and generalizability of the findings. Future prospective studies with standardized protocols and larger cohorts are needed to address these issues more robustly.

A potential strength is the inclusion of a heterogeneous patient population representative of routine clinical practice, which allows for a more realistic assessment of the effectiveness of excision and ablation in everyday conditions. Additionally, the extended follow-up period enables the evaluation of long-term developments, particularly with regard to symptom recurrence and the necessity of repeat surgery.

In conclusion, this retrospective study demonstrates the clinical value of both excision and ablation as surgical options for the treatment of peritoneal endometriosis, with postoperative improvements in pain outcomes observed across both groups. While no clear superiority of one technique over the other could be identified, the findings suggest that long-term symptom control, particularly for dysmenorrhea, is more strongly influenced by postoperative management and time since surgery than by the choice of surgical technique. Given the heterogeneity of patient presentations and the chronic nature of the disease, individualized treatment strategies remain essential. The study further highlights the need for prospective, high-quality trials with standardized surgical techniques, comparable subgroups, and uniform follow-up protocols to better delineate optimal approaches for symptom control, fertility preservation, and prevention of recurrence in patients with endometriosis.

## Supplementary Information

Below is the link to the electronic supplementary material.

Supplementary file1 (DOCX 28 KB)

## Data Availability

The datasets used and analyzed during the study are available from the corresponding author on request.
