# Supplementary material for: Ablation compared with excision in the surgical management of peritoneal endometriosis: a retrospective study of pain, re-operation, and pregnancy outcomes
Source: Arch Gynecol Obstet. 2026 Feb 2;313(1):73. doi: 10.1007/s00404-026-08331-4 (PMC12864340; doi:10.1007/s00404-026-08331-4)
Supplement: Supplementary file 1 — Supplementary file1 (DOCX 28 KB) [file 404_2026_8331_MOESM1_ESM.docx]

| **Dysmenorrhea** | | | | | | | | | |  |
| --- | --- | --- | --- | --- | --- | --- | --- | --- | --- | --- |
| a) Random effects | | | | | | | | | |  |
| Parameter | | n | | | Coefficient | SE | | 95% CI | |  |
| SD (Intercept: Patient ID) | | 67 | | | 1.95 | 0.28 | | [1.47, 2.58] | |  |
| SD (Residual) | | 154 | | | 2.99 | 0.15 | | [2.71, 3.30] | |  |
| b) Fixed effects | | | | | | | | | | |
| Variable | Coefficient | | SE | 95% CI | | | t-value | | p-value | |
| Intercept | 5.48 | | 0.53 | [4.43, 6.53] | | | 10.28 | | < .001 *** | |
| Surgical technique (Ablation = baseline) | -0.53 | | 0.62 | [-1.75, 0.70] | | | -0.84 | | 0.400 | |
| Hormonal Therapy | -1.13 | | 0.47 | [-2.06, -0.20] | | | -2.40 | | 0.017 * | |
| Time since surgery | -0.06 | | 0.0098 | [-0.08, -0.04] | | | -6.07 | | < .001 *** | |
|  | | | | | | | | | | |
| **Dyspareunia** | | | | | | | | | |  |
| a) Random effects | | | | | | | | | |  |
| Parameter | | n | | | Coefficient | SE | | 95% CI | |  |
| SD (Intercept: Patient ID) | | 67 | | | 1.35 | 0.13 | | [1.11, 1.63] | |  |
| SD (Residual) | | 154 | | | 0.90 | 0.05 | | [0.82, 0.99] | |  |
| b) Fixed effects | | | | | | | | | | |
| Variable | Coefficient | | SE | 95% CI | | | t-value | | p-value | |
| Intercept | 0.23 | | 0.29 | [-0.34, 0.80] | | | 0.79 | | 0.432 | |
| Surgical technique (Ablation = baseline) | 0.37 | | 0.36 | [-0.34, 1.08] | | | 1.03 | | 0.302 | |
| Hormonal Therapy  (no therapy = baseline) | 0.22 | | 0.16 | [-0.09, 0.54] | | | 1.40 | | 0.163 | |
| Time since surgery | 0.0062 | | 0.0030 | [-0.01, 0.00] | | | -2.08 | | 0.038 | |
|  | | | | | | | | | | |
| **Chronic pelvic pain** | | | | | | | | | |  |
| a) Random effects | | | | | | | | | |  |
| Parameter | | n | | | Coefficient | SE | | 95% CI | |  |
| SD (Intercept: Patient ID) | | 67 | | | 1.35 | 0.13 | | [1.11, 1.63] | |  |
| SD (Residual) | | 154 | | | 0.90 | 0.05 | | [0.82, 0.99] | |  |
| b) Fixed effects | | | | | | | | | | |
| Variable | Coefficient | | SE | 95% CI | | | t-value | | p-value | |
| Intercept | 1.03 | | 0.46 | [0.12, 1.94] | | | 2.22 | | 0.027 * | |
| Surgical technique (Ablation = baseline) | 1.10 | | 0.56 | [-0.01, 2.20] | | | 1.96 | | 0.051 | |
| Hormonal Therapy  (no therapy = baseline) | -0.33 | | 0.33 | [-0.97, 0.32] | | | -1.00 | | 0.321 | |
| Time since surgery | -0.02 | | 0.0064 | [-0.03, -0.01] | | | -3.52 | | < .001 *** | |

**Supplement Table 1**: The table shows the mixed effects models of the three pain modalities: dysmenorrhea, dyspareunia, and chronic pelvic pain length. For each patient, her random effect is considered as the realization of independent normally distributed random variables with zero mean and unknown standard deviation (SD), whose estimate is given in the respective sub-tables a). These random effects may be considered as the patient’s individual deviation from a common intercept. Similarly, the residual errors are assumed to be normally distributed with zero mean and common standard deviation (SD). In the sub-tables b), the fixed effects of the three variables: surgical technique, hormonal therapy, and time since surgery, are shown. The weights are given in the coefficient column. Significant results are marked with asterisks (p < 0.05: *, p < 0.001: ***). SE: standard error, CI: confidence intervall.

| **Patient ID** | **Surgical technique** | **Month until next surgery** | **Commentary** |
| --- | --- | --- | --- |
| 6 | Excision and ablation | 16 | Resection of peritoneal endometriosis |
| 7 | Excision and ablation | 8 | Resection of peritoneal endometriosis |
| 14 | Excision | 20 | NovaSure endometrial ablation |
| 15 | Excision | 24 | Resection of deep infiltrating endometriosis |
| 19 | Excision | 12 | 2019: Resection of peritoneal endometriosis and adenomyosis, 2021: Hysterectomy |
| 21 | Excision | 28 | Resection of peritoneal endometriosis |
| 31 | Excision | 39 | Hyserectomy |
| 32 | Excision | 22 | Resection of peritoneal endometriosis |
| 53 | Excision | 21 | Hysterectomy |
| 58 | Excision | 24 | Resection of deep infiltrating endometriosis of the bowel |
| 60 | Ablation | 33 | Resection of peritoneal endometriosis |
| 94 | Excision | 24 | Resection of peritoneal endometriosis |

**Supplement Table 2:** This table presents the selected patients who required an additional surgery. It includes the surgical technique used, the number of months between the primary and subsequent surgery, and the type of procedure performed.
